# COMPARATIVE STUDY OF MICROANASTOMOSIS WITH DISTINCT 10-0 NYLON SUTURES IN RATS

**DOI:** 10.1590/1413-785220162401152773

**Published:** 2016

**Authors:** Ricardo Teixeira e Silva, Thiago Felipe Santos Barros, José Thomé de Carvalho, André Araújo Ribeiro, André Fernandes Pires, Teng Hsiang Wei

**Affiliations:** 1. Universidade de São Paulo, Faculdade de Medicina, Hospital das Clínicas, Instituto de Ortopedia e Traumatologia, São Paulo, SP, Brazil.

**Keywords:** Anastomosis, sugical, Microsurgery, Rats, Wistar, Sutures

## Abstract

**Objective:**

The aim of this study is to compare micro-sutures commonly used in our midst.

**Methods:**

In this double-blind study, 30 Wistar rats were operated randomly divided into three groups matched according to the suture used (Nylon 10-0, 75micron, brands Microsuture^(r)^, Polysuture^(r)^ and Ethicon^(r))^. We analyzed the number of surgical nodes required, bleeding, surgical time and histological evaluation.

**Results:**

There was no significant difference between the amount of stitches of arterial suture per anastomosis. Surgical time was longer in Microsuture^(r)^ group as compared to Polysuture^(r)^ (p ≤ 0.05). Bleeding in Microsuture^(r)^ group was higher when compared to the others (p <0.01). In the histological analysis, the Microsuture^(r)^ group showed a greater tendency to develop fibrosis and aneurysm in surgical site than the others (p <0.01 and p≤0,05, respectively). Similarly, the Ethicon^(r)^ group showed less tendency to myointimal proliferation than the rest. (p = 0.025).

**Conclusion:**

The results confirm the relevance of the choice of surgical thread as an independent determining factor for the success of the procedure, besides serving as a rational subsidy for a better cost-benefit analysis.*** Level of Evidence I, Experimental Study, Controlled Animal Study.***

## INTRODUCTION

Microsurgery is becoming indispensable in many areas of modern medicine, such as plastic surgery, hand surgery, and otorhinolaryngology, among others. Thanks to the technological development of materials and instrumental, it has become increasingly accurate and consistent.

Microsurgical vascular suture is an essential step that takes time, besides the quality of materials and instrumental, that are essential for obtaining the expected outcomes.^1^

The microsurgical procedure, even in experienced hands and performed with atraumatic technique, causes some degree of injury to the vessel wall, due to its transfixing through the needle and suture nodes. Taking into consideration that the suture wires are of fundamental importance at this stage and subsequent surgical outcome, the aim of this study was to evaluate the 10-0 size nylon suture brands used in microvascular surgery in Brazil and their relationship to the procedure outcomes.^2^

## METHODS

Within five months we operated 30 Wistar rats randomly divided into three groups according to the suture brand of 0.75 microns Nylon 10: Microsuture^(r)^ (Microsuture Ltd, Brazil), Polysuture^(r)^

(Brazil) and Ethicon^(r)^ (Ethicon US, LLC, USA) used by the surgeon, who was not aware of which wire brand he was using (blinding). The sample size was determined at the beginning of the study through specific statistical tests. The risk management standards œ (5%) and ß (20%) were considered, as well as the diversity of variables. We, thus, reached a minimum of 30 surgical procedures.

Considering the fact that one of the largest research limiters in microanastomoses in laboratory animals are surgical failure and the death of animals, we chose the protocol proposed by Schleimer et al.^3^ In this study, the researchers obtained excellent results regarding animals survival and also functionality of the sutures. The study sequence, which comprised the choice of animals, anesthesia, surgical preparation, surgical technique, post-procedural care and analysis of the results, as described in the following paragraphs, was based on such protocol.

All animals (30 white Wistar rats, weighing on average 350g) were obtained from the animal facility of *Faculdade de Medicina da Universidade de São Paulo*, SP, Brazil, after approval by the Ethics Committee for Animal Experimentation (IOT Protocol N° 1030), in accordance with current legislation.

The rats were kept in a vivarium in cages with water and food *ad libitum*. They underwent surgery in the laboratory of microsurgery under proper technical supervision. The animals were anesthetized with intramuscular xylazine 10 mg/kg plus ketamine 50-75 mg/kg. We used three brands of 75 micron nylon 10-0 suture wire: Microsuture^(r)^, Polysuture^(r)^ and Ethicon^(r)^. 

After anesthesia, the animals were shaved and placed in horizontal supine position. Antisepsis was made in the groin and medial thigh region. Then, a transverse incision was made in the thigh following the inguinal crease. The subcutaneous tissue was dissected toward the skin incision and epigastric vessels were cauterized and the femoral neurovascular bundle was identified. The femoral artery was individualized via dissection of the connective tissue that connects it to other structures. A rubber shield was used for better visualization of the femoral artery. The vessel was flushed with 2% lidocaine to prevent arterial spasm. A double clamp was positioned between the inguinal ligament and the beginning of the inferior epigastric branches (distally) and the arteriotomy was held at half distance from the double clamp. Then, stumps were prepared by removal of the adventitious, dilation with dilating forceps and lumen irrigation with 5000 IU/ml sodium heparin.

The clamps were approached and then sutured with interrupted sutures through the triangulation technique (two anterior points, 120° between them). The clamp was turned facing the rear wall suture, and the anterior wall suture was ended with 8-10 stitches on average. By the end of the suture, the clamp was removed and placed in precision balance pre-weighed gauze for 3 min. After that, the gauze was weighed once again to quantify the anastomosis bleeding by subtracting the final weight of the gauze from its former weight. At this time, the anastomosis patency was tested. The closure of the incision was performed through simple suture with non-absorbable nylon 4-0 wire.

After 10 days postoperatively, on average, when animals were supervised by a veterinarian, the rats were subjected to a new intervention for anastomoses rapprochement. The anesthetic procedure was performed as in the first surgery. The skin stitches were removed and the dissection and individualization of the femoral arteries was held according to previously described procedures.

The final anastomosis permeability test was performed using the distal section to the blood vessel anastomosis and direct observation of presence of blood flow, being expected pulsating bleeding of the artery in question.

The femoral artery was connected by two ends and a segment of arterial anastomosis was resected. The lumen of the vascular segments was irrigated with a 10% formaldehyde solution with a syringe and a jelco catheter. The animals, still anesthetized, were euthanized by intravenous lethal injection, following the 1999 recommendations of *Colégio Brasileiro de Experimentação Animal* (COBEA). The vascular segments were anchored to a small piece of synthetic foam by its edges using microsurgical needles in order to avoid vessel deformation.

The vessels segments were sent to the Anatomopathological Service. After proper processing, the surgical specimens were fully included in paraffin blocks. The cuts were performed every 3 µm in the longitudinal plane of the vessels. Each sample was evaluated by three staining methods: hematoxylin-eosin (HE), Masson's trichrome, and Verhoff's. The slides were blind examined by the same pathologist in a conventional optical Zeiss^(r)^ microscope using 50-200 fold augmentation. The perivascular inflammation was ranked in each sample as absent, mild, moderate or intense. Furthermore, samples were assessed for the presence of aneurysm or thrombus. All slides were blind evaluated by an experienced pathologist.

The Laboratory of Medical Investigation of the Musculoskeletal System of *Faculdade de Medicina da Universidade de São Paulo*, with its funds, has borne the resources to carry out the necessary procedures.

For characterization of the quantitative data descriptive statistics of parameters measured was performed: mean, standard deviation, minimum and maximum values. 

To compare the test results regarding the histological analysis of anatomical parts, divided into specific classes, the Pearson's chi-square test was employed.

The normality test for weight, length of surgery, number of nodes and bleeding weight was the Kolmogorov-Smirnova test. 

In all cases we adopted 5% significance level (α=0.05).

## RESULTS

There was no difference on the average weight of the rats in the three groups analyzed, namely Microsuture^(r)^, Polysuture^(r)^ and Ethicon^(r)^ groups, confirming the pairing of the groups. There was also no significant difference between the quantities of stitches of arterial anastomosis suture in each of the three groups analyzed.

The time required to complete each arterial anastomosis was 37.36 min, on average, in the Microsuture^(r)^ group, 32.40 min in the Polysuture^(r)^ group and 32.56 min in the Ethicon^(r)^ group. The difference between values was statistically significant (p ≤ 0.05) when comparing the Microsuture^(r)^ group with the Polysuture^(r)^ group, the latter showing a lower surgical time. There was no significant difference between the other pairs. (Table 1)

**Table 1:** Distribution of anastomosis time among groups.

Microsuture^(r)^	Polysuture^(r)^	Ethicon^(r)^
Mean (minutes)	37.36	32.40	32.56
Standard deviation	5.537	2.757	4.746
Standard error	1.669	0.872	1.582

Post hoc test (p ≤ 0.05).

The bleeding occurred in each procedure, quantified as previously described, was higher in the Microsuture^(r)^ group as compared to Polysuture^(r)^ and Ethicon^(r)^ groups, with no difference between the last two. (Table 2)

**Table 2:** Distribution of bleeding among groups.

Microsuture^(r)^	Polysuture^(r)^	Ethicon^(r)^
Mean (g)	1.3570	0.4718	0.2050
Standard deviation	0.69664	0.36471	0.17296
Standard error	0.22030	0.10997	0.05470

Post hoc test (p ≤ 0.01).

Histological analysis were ranked as absent, mild, moderate or intense in each sample, except for the dichotomized variables analysis such as thrombus and aneurysm, which were discriminated as present or absent.

There was no difference in the degree of perivascular inflammatory process among the three groups analyzed.

Statistically significant differences were not observed between the groups regarding the foreign body perivascular granulomatous reaction, as well as vascular proliferation.

There was only one identified case of intravascular thrombus on all slides analyzed, which belonged to the Microsuture^(r)^ group, without statistical validity.

There was a statistically significant difference regarding perivascular fibrosis between the groups. Seventy percent of cases sutured with Microsuture^(r)^ showed moderate fibrosis at the surgical site, while 90% of rats sutured with Polysuture^(r)^ or Ethicon^(r)^ showed absent or mild fibrosis. (Table 3)

**Table 3:** Distribution of perivascular fibrosis degree among groups.

Microsuture^(r)^	Polysuture^(r)^	Ethicon^(r)^
Absent	1	0	2
Discrete	2	9	7
Moderate	7	1	1
Intense	0	0	0
N=10	N=10	N=10

Pearson's Chi-square test (p< 0.01).

Three among all rats showed aneurysm in the artery submitted to anastomosis, solely in the Microsuture^(r)^ group. This outcome distribution provided statistical significance to the fact. (Table 4)

**Table 4:** Presence or absence of aneurisms on arterial anastomosis among groups.

Microsuture^(r)^	Polysuture^(r)^	Ethicon^(r)^
Absent	7	10	10
Present	3	0	0
N=10	N=10	N=10

Pearson's Chi-square test (p< 0.01).

Finally, a statistically significant difference between the groups was observed when analyzing the proliferation of the arterial intimal layer. Sixty percent of the rats in the Ethicon^(r)^ group had no myointimal proliferation. Ninety percent of the cases sutured with Microsuture^(r)^ and 80% of those sutured with Polysuture^(r)^ presented a discrete level of proliferation. (Table 5) 

**Table 5:** Distribution of degree of proliferation of the arterial intimal layer among groups.

Microsuture^(r)^	Polysuture^(r)^	Ethicon^(r)^
Absent	0	2	6
Discrete	9	8	3
Moderate	1	0	1
Intense	0	0	0
N=10	N=10	N=10

Pearson's Chi-square test (p= 0.025).

## DISCUSSION

The surgical procedure involves many variables that can change its outcome, and the properties of the suture wire is one of them. Thus, it is necessary to evaluate the various suture wires used in surgical practice and their implications.

Although many of the evaluated aspects did not show significant differences, some vital aspects to favorable outcomes showed significant discrepancies.

There was a statistically significant difference (p ≤ 0.05) regarding the average of surgical time (time measured from skin incision to closure), between the groups; Microsuture^(r)^ group showed a longer average surgical time than Polysuture^(r)^, increasing, thus, stress and surgical risk.

Moreover, in microsurgery, the ischemic time that a tissue is subjected is inversely proportional to its survival and to the likelihood of reversing to an unfavorable situation and tissue necrosis.^4^ ^,^ ^5^

Regarding the amount of bleeding, we noticed noted that Microsuture^(r)^ group showed a statistically significant (p <0.01) average bleeding mass (1.357g) higher than the other groups, which may determine poorer surgical outcomes.

It is possible to associate that fact to variables such as trauma caused by the material, wire tensile strength, needle standardization, among other essential biomechanical factors to perform the appropriate technique.^6^

Despite the histological analysis did not show statistically significant differences regarding the degree of perivascular inflammatory process, foreign body perivascular granulomatous reaction, and vascular proliferation, it was positive in other respects.

Perivascular fibrosis is part of the proliferative stage of wound healing physiology, and many factors influence this process, including immune status, degree of contamination, age, and mainly the type of the primary injury.^4^ Seventy percent of cases sutured with Microsuture^(r)^ wire showed moderate fibrosis in the surgical site, while 90% of rats sutured with Polysuture^(r)^ or Ethicon^(r)^ showed absent or mild fibrosis (statistically significant results). Therefore, the type of primary injury caused by the suture wire and its physical characteristics strongly influences the vessel healing process.

The ideal wire, therefore, must be resistant to tension and torsion in its environment; show uniform caliber and low friction index; not cutting; resistant to sterilization; good node safety and low tissue reaction.^7^ It has already been proposed that health services should promote the management of suture wires in order to ensure the quality, effectiveness and safety of technologies and materials used in health care, reporting any non-compliance to the National Health Surveillance System.

## CONCLUSION

The results of this study corroborate the relevance of the surgical wire choice as an independent determining factor for the success of the procedure. Therefore, they may support a rational choice of suture wires in medical practice, depending on cost, effectiveness and safety.
